# A SPECTRUM OF AGING: INTRODUCTION OF A REFLECTED EXPERIENCE MODEL OF AGING WELL

**DOI:** 10.1093/geroni/igae098.4105

**Published:** 2024-12-31

**Authors:** Patricia Davis Chilton, Priscilla Magrath, Matthew Grilli, Valeria Pfeifer, Daniel Sullivan, Matthias Mehl

**Affiliations:** University of Arizona, Tucson, Arizona, United States; University of Arizona, Tucson, Arizona, United States; University of Arizona, Tucson, Arizona, United States; University of Arizona, Tucson, Arizona, United States; University of Arizona, Tucson, Arizona, United States; University of Arizona, Tucson, Arizona, United States

## Abstract

Current methods to study aging lack a personalized approach in investigating the question of what it means to age well - the narrated, lived experience of those doing the aging. The current study collected narratives from 92 individuals between the ages of 62 and 95. Participants were interviewed via Zoom Health and provided four minutes of spontaneous (i.e. unprepared) answers to the question, “In your own words, what does aging well mean to you?”. The responses were recorded, transcribed using speech-to-text, deidentified, and checked for accuracy by trained research assistants. The responses were analyzed qualitatively using an interpretivist approach, inductively identifying recurring ideas from the narratives, and extracting themes that represent a broad spectrum of aging well experiences and meaning. The main themes that emerged were: Lifestyles/Health/Wellness, Compensation/Adaptation, Social Connection, Social Contribution, Existential, and Time Awareness. A thematic map was created representing the main themes and their 24 subthemes. Using MAXQDA software, a descriptive analysis of the coded themes indicated that out of 1,618 coded segments, 28% represented Lifestyle/Health/Wellness themes (e.g. physical/cognitive health, travel, finances), 25% reflected Existential themes (e.g. purpose/meaning, emotional wellbeing, spirituality, openness/growth), 15% represented Social Connection, 10% were Compensation/Adaptation (e.g. interdependence, technology, adjusting environment/mindset), while 6% represented both social contribution (e.g. volunteerism) and time awareness. These analyses constitute a first step towards a reflected experience model of aging and can facilitate a richer understanding of the diversity and lived experience of aging well.

